# Aloe-emodin: from pharmacological mechanisms to clinical applications and future perspectives

**DOI:** 10.3389/fphar.2025.1741679

**Published:** 2026-01-12

**Authors:** Jin Xie, Junfeng Zhang, Xiaoyan Chen

**Affiliations:** 1 Hubei University of Chinese Medicine, Wuhan, China; 2 Department of rehabilitation, Taihe Hospital, Hubei University of Medicine, Shiyan, China; 3 School of Basic Medical Sciences, Hubei University of Chinese Medicine, Wuhan, China

**Keywords:** aloe-emodin, anti-inflammatory effects, antitumor, bioavailability, combination therapy, drug delivery, pharmacological mechanisms

## Abstract

Natural products continue to be fundamental to contemporary drug discovery. Aloe-emodin, a natural anthraquinone molecule sourced from plants including aloe and rhubarb, has attracted considerable interest owing to its diverse pharmacological properties. This review analyzes the complex modes of action of AE, emphasizing its significant anti-cancer effects by targeting critical signaling pathways like PI3K/Akt, MAPK, and NF-κB, which induce apoptosis and cell cycle arrest, regulate autophagy, and inhibit metastasis. In addition to oncology, AE exhibits potent anti-inflammatory, neuroprotective, and antiviral effects primarily by reducing oxidative stress and regulating inflammatory responses. Notwithstanding its encouraging preclinical performance, the practical application of AE has been impeded by considerable obstacles, notably its inadequate bioavailability, possible toxicity, and absence of target selectivity. We rigorously assess these challenges and examine novel tactics designed to improve its therapeutic index, including nanotechnology-based drug delivery devices and alterations to chemical structures. This review seeks to reconcile the intricate pharmacology with clinical applicability, offering a prospective outlook on the use of AE as a next-generation therapeutic agent for cancer and other complex disorders.

## 1 Introduction

Since antiquity, nature has served as an exceptional reservoir, containing a vast array of structurally varied and physiologically active compounds that have significantly influenced the overarching framework of contemporary drug development (Wang M. et al., 2025). Natural products, such as the antimalarial agent artemisinin and the anticancer medicine paclitaxel, have consistently yielded innovative chemical frameworks and lead compounds for drug development, perpetually fostering innovation within the pharmaceutical sector (Atanasov et al., 2021; Li et al., 2022). The anthraquinone family is distinguished by its tricyclic aromatic quinone core structure and extensive range of pharmacological actions. These chemicals are extensively found in plants, fungus, and lichens and has a longstanding history of use in traditional medicine (Mustafa et al., 2025; Olszewski et al., 2024). Aloe-emodin (AE), a hydroxyanthraquinone, is gaining prominence in contemporary scientific research, stemming from its historically established use.

Aloe-emodin, or 1.8-dihydroxy-3-hydroxymethylanthraquinone, is a prevalent active compound found in several medicinal plants, including the rhizomes of Rheum species and the leaves of Aloe species (Yan et al., 2023; Pecere et al., 2021). Its peculiar planar aromatic configuration, enhanced by particular hydroxyl and hydroxymethyl alterations, confers unique physicochemical characteristics, allowing it to engage with many biological targets (Wang Z. et al., 2025; Lin et al., 2022). Historically recognized mainly for its laxative properties, recent research over the past 20 years has started to uncover its more intricate and fascinating pharmacological profile. This evolution from traditional medicine to a molecule with considerable therapeutic promise signifies a quintessential renaissance of a natural product in contemporary science (Ouyang et al., 2024).

The renewed scientific interest in aloe-emodin arises from a shift in medication development paradigms. As our comprehension of intricate multifactorial diseases including cancer, neurological disorders, and chronic inflammation advances, the conventional “single target, single drug” research and development paradigm has revealed its constraints (Bi et al., 2025; Ragab et al., 2023). The scientific community increasingly acknowledges that multi-target medicines, which can concurrently modulate numerous interconnected signaling networks, possess significant potential. Aloe-emodin exemplifies such compounds (Qu et al., 2025; Hu et al., 2023). The swift increase in relevant research publications during the early 21st century indicates widespread acknowledgment of its capacity to modulate several biological processes, including as cell proliferation, apoptosis, autophagy, and inflammation (Chen et al., 2023). This pleiotropy positions aloe-emodin as a formidable contender for addressing complex disorders marked by dysregulated pathogenic networks.

Nonetheless, despite its significant potential, the transition from a preclinical lead chemical to a clinical medicine for aloe-emodin is laden with obstacles. The extensive target profile prompts several essential inquiries: What is the complexity of its mechanism of action? Does this pleiotropy confer a therapeutic advantage or result in off-target toxicity? Furthermore, its inadequate water solubility and diminished oral bioavailability provide considerable challenges to clinical application (He et al., 2023; Ren et al., 2024). This review explores the intricate biological properties of aloe-emodin, analyzes its fundamental pharmacological mechanisms, especially its anti-tumor and anti-inflammatory effects, evaluates its therapeutic potential across diverse disease models, identifies significant barriers to its clinical application, and underscores novel strategies devised to surmount these challenges, with the objective of offering a thorough and progressive outlook on the transformation of this ancient molecule into next-generation precision therapeutic agents.

## 2 The pharmacological mechanism of aloe-emodin

### 2.1 Antitumor mechanism

#### 2.1.1 Induction of cell cycle arrest and apoptosis

**FIGURE 1:**
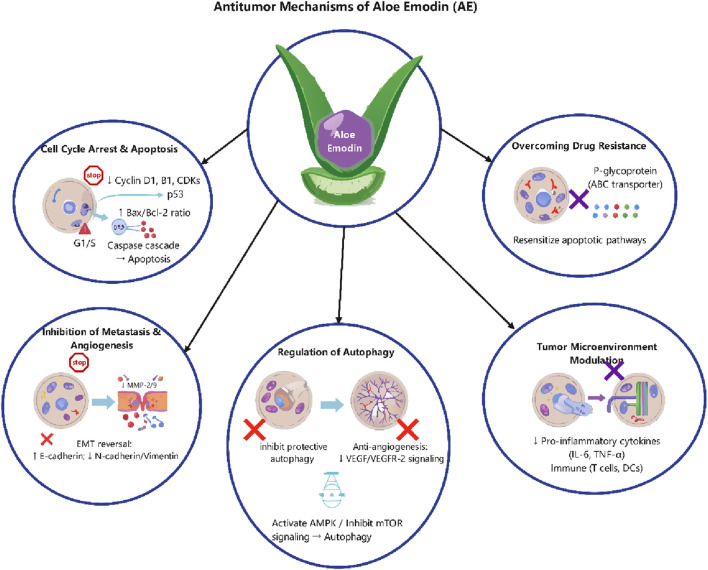
The multifaceted antitumor mechanisms of AE. AE exerts its anticancer effects through five primary pathways.

A primary anti-cancer strategy of AE is to halt the continuous proliferation of cancer cells by inducing cell cycle arrest (Zhou et al., 2024). It primarily accomplishes this by down-regulating the expression or activity of cell cycle proteins, including Cyclin D1, B1, and their cyclin-dependent kinases (CDKs), so obstructing the cell cycle at the G1/S or G2/M transition stages (Kung et al., 2025; Witkiewicz et al., 2022; Niu et al., 2023). Concurrently, AE is a powerful inducer of apoptosis. It can concurrently activate both the internal mitochondrial and extrinsic death receptor apoptotic pathways (Zhang W. et al., 2023). It often enhances the expression of the tumor suppressor protein p53, thereby modulating the Bcl-2 protein family by elevating the ratio of pro-apoptotic members, such as Bax and Bak, to anti-apoptotic members, such as Bcl-2 and Bcl-xL (Hao et al., 2023; Wei et al., 2023; Wei et al., 2021). This disturbs the mitochondrial membrane potential, resulting in the release of cytochrome c, which then activates the Caspase protease cascade, thus executing the cell death program (Zhang X. et al., 2023) (Figure 1).

#### 2.1.2 Bilateral regulation of cellular autophagy

Autophagy is a cellular self-digestion mechanism that serves a context-dependent dual function in cancer. The relationship between autophagy and AE is similarly intricate, like to a “double-edged sword”. In certain cancer types, AE can trigger cytotoxic autophagy, resulting in autophagic cell death and thereby amplifying its anti-cancer efficacy (Huang et al., 2021; Li R. et al., 2025; Wu JJ. et al., 2024). Under different conditions, it may impede protective autophagy, which cancer cells employ to withstand stress and chemotherapy (Huang et al., 2023; Zhu et al., 2021). Consequently, the inhibition of autophagy can enhance the susceptibility of cancer cells to alternative therapies. The molecular switches governing this duality often encompass the AMP-activated protein kinase (AMPK) and mammalian target of rapamycin (mTOR) signaling pathways (Wang H. et al., 2022; Bahar et al., 2024; Xu et al., 2025). Activation of AMPK or inhibition of mTOR by AE can induce autophagy; nevertheless, the fate of the cells whether they perish or endure depends on the particular cellular milieu and the magnitude of the autophagic flux (Paquette et al., 2021).

#### 2.1.3 Inhibition of tumor metastasis and angiogenesis

The predominant cause of cancer-related mortality is tumor metastasis. AE exhibits considerable anti-metastatic efficacy by targeting critical phases in the metastatic cascade (Qu et al., 2025; Jiang et al., 2021; Şeker et al., 2022). Research indicates that it can inhibit the function of matrix metalloproteinases (MMPs), specifically MMP-2 and MMP-9, which are essential for extracellular matrix degradation and facilitating cancer cell invasion (Şeker et al., 2022). Furthermore, AE can reverse the epithelial-mesenchymal transition, a process wherein cancer cells lose epithelial traits and gain migratory and invasive capabilities. AE accomplishes this reversal by up-regulating epithelial markers such as E-cadherin while down-regulating mesenchymal markers like N-cadherin and Vimentin (Chen et al., 2023). Additionally, AE impedes tumor growth by inhibiting angiogenesis, primarily by suppressing the expression and signaling of vascular endothelial growth factor (VEGF) and its receptor VEGFR-2 (Hassan et al., 2024; Dai et al., 2025).

#### 2.1.4 Bilateral modulation of the tumor microenvironment

The tumor microenvironment (TME) is a multifaceted ecosystem consisting of cancer cells, immune cells, fibroblasts, and signaling molecules, which significantly affects tumor growth and therapeutic response (Chen X. et al., 2025). AE is becoming a potent modulator of TME. It can “re-educate” pro-tumorigenic M2 type tumor-associated macrophages to polarize into the anti-tumor M1 phenotype (Shah et al., 2022). This change aids in reactivating local anti-cancer immune responses (Li X. et al., 2024). Moreover, by inhibiting pro-inflammatory cytokines like IL-6 and TNF-α in the TME, AE can mitigate chronic, tumor promoting inflammation and modulate the activities of other immune cells, including T cells and dendritic cells, thus establishing a microenvironment detrimental to tumor proliferation (Qu et al., 2025; Yin et al., 2025).

#### 2.1.5 Overcoming drug resistance

Acquired or intrinsic medication resistance is a significant challenge in cancer chemotherapy. AE, as a chemical sensitizer, has demonstrated significant potential in restoring or augmenting the potency of conventional anticancer agents (Cheng et al., 2021). A primary mechanism is the inhibition of ATP binding cassette transporters, including P-glycoprotein, which function as “drug pumps” to extrude chemotherapeutic drugs from cancer cells (Yoganathan et al., 2021; Moinul et al., 2022; Sun et al., 2025). By inhibiting these pumps, AE enhances the intracellular retention of medicines like as cisplatin, doxorubicin, and paclitaxel (Hanssen et al., 2021; Liang et al., 2021). It can resensitize drug resistant cells by modulating the dysregulated signaling pathways that contribute to resistance, therefore reinstating their apoptotic capability (Bharathiraja et al., 2023). This renders AE a particularly effective adjunctive medication in combination therapy protocols (Liang et al., 2021).

### 2.2 Anti-inflammatory and immunomodulatory effects

#### 2.2.1 Targeting key inflammatory pathways

The anti-inflammatory efficacy of AE is primarily ascribed to its potent suppression of various established pro-inflammatory signaling pathways. It can effectively inhibit the activation of NF-κB, a principal transcription factor that governs the expression of several inflammatory genes (Wang WB. et al., 2022; Shang et al., 2022). AE generally sequesters NF-κB in the cytoplasm by inhibiting the degradation of the NF-κB inhibitor IκBα (Li H. et al., 2025; Shen et al., 2024). Furthermore, it can obstruct the phosphorylation and activation of the mitogen-activated protein kinase (MAPK) pathway, encompassing p38, ERK, JNK, and the Janus kinase/signal transducer and activator of transcription (JAK/STAT) pathway, both of which are pivotal in orchestrating inflammation and immune responses (Lu et al., 2025).

#### 2.2.2 Reduce oxidative stress

Oxidative stress, resulting from the disparity between reactive oxygen species (ROS) production and the cellular antioxidant defense mechanism, is intricately linked to inflammation (Song et al., 2022). AE functions not only as a direct ROS scavenger but also exerts a significant antioxidant effect indirectly (Ren et al., 2024). The primary indirect method is the stimulation of the nuclear factor erythroid 2-related factor Nrf2 pathway (He et al., 2023). AE facilitates the translocation of Nrf2 to the nucleus, consequently upregulating the expression of various antioxidant and cytoprotective genes, including heme oxygenase-1 and NAD(P)H:quinone oxidoreductase 1(NQO1), which enhances the cellular intrinsic defense against oxidative damage (Ouyang et al., 2024).

### 2.3 Other emerging pharmacological activities

#### 2.3.1 Neuroprotective effect

In the domain of neurodegenerative disorders, AE has exhibited encouraging neuroprotective properties (Yan et al., 2023). In preclinical Alzheimer’s disease models, it has been shown to impede the aggregation of β-amyloid protein and diminish the hyperphosphorylation of Tau protein, both of which are critical pathological indicators of the disease (Li X. et al., 2024). In Parkinson’s disease models, AE can safeguard dopaminergic neurons from neurotoxin-induced apoptosis, partially due to its robust anti-inflammatory and antioxidant effects, which mitigate the chronic neuroinflammation propelling disease advancement (Mitra et al., 2022; Xian et al., 2021).

#### 2.3.2 Antiviral and antibacterial effects

AE has broad-spectrum antimicrobial activity. It has been proven effective against a range of enveloped viruses, including influenza virus, herpes simplex virus, coronavirus, etc (Wu J. et al., 2024). Its mechanism of action is usually to interfere with the virus’s entry, attachment or replication process (Li Z. et al., 2024). Its antibacterial effect has been reported in both Gram-positive and Gram-negative bacteria. Possible mechanisms include disrupting the bacterial cell membrane, inhibiting nucleic acid synthesis, and interfering with the bacterial quorum sensing system (Hu et al., 2023).

#### 2.3.3 Metabolic regulatory function

Initial research indicates that AE may have a positive impact on metabolic disorders. In animal models of type 2 diabetes and obesity, AE has demonstrated enhancement of insulin sensitivity, reduction of blood glucose levels, and mitigation of lipid buildup in hepatic and adipose tissues (Ouyang et al., 2024). Certain effects are thought to be facilitated by the activation of AMPK, a fundamental regulator of cellular energy metabolism, which intersects with its mechanisms in cancer and autophagy (Bao et al., 2024; Yu et al., 2023). These findings present a novel opportunity for investigating the application of AE in the management of metabolic syndrome.

## 3 Clinical potential and therapeutic applications

### 3.1 Preclinical evidence in disease models

The therapeutic efficacy of AE has been validated in multiple preclinical animal models encompassing oncology, inflammation, and neurology. In many cancer xenograft models, including lung cancer (Kim et al., 2022), breast cancer (Cheng et al., 2021), and glioblastoma (Fang et al., 2023), the administration of AE, either alone or in conjunction with other treatments, markedly suppresses tumor development, diminishes tumor volume, and extends survival. These effects are frequently associated with molecular alterations observed *in vitro* and *in vivo*, including an elevation in apoptotic markers such as cleaved Caspase-3 and a reduction in proliferative indicators like Ki-67^36^. In models of inflammatory diseases, including collagen-induced arthritis and lipopolysaccharide-induced acute lung injury, AE reliably mitigates tissue damage, diminishes inflammatory cell infiltration, and decreases circulating pro-inflammatory cytokine levels (Qu et al., 2025; Deng et al., 2025). Furthermore, in models of Parkinson’s disease generated by neurotoxins or transgenic models of Alzheimer’s disease, AE exhibits the capacity to safeguard neurons, enhance motor or cognitive functions, and diminish neuroinflammatory markers (Yan et al., 2023; Li X. et al., 2024) (Table 1).

**TABLE 1:** Preclinical efficacy of aloe-emodin in various animal models of disease.

Therapeutic area	Disease model	Animal species	Dosing regimen	Key observed outcomes	Mechanistic findings
Oncology	Lung cancer	Nude mice	50 mg/kg, intraperitoneal injection	Inhibit tumor growth and tumor angiogenesis; reduce tumor cell proliferation	Induction of apoptosis; upregulation of cleaved Caspase-3 and Bax; downregulation of Bcl-2; inhibition of proliferation: downregulation of Ki-67 and PCNA expression; inhibition of VEGF expression (Kim et al., 2022)
	Breast cancer	Nude mice	50 mg/kg, intraperitoneal injection	Significantly inhibit tumor growth and volume; inhibit tumor metastasis	Induce apoptosis of cells; inhibit the Akt/mTOR signaling pathway; inhibit the activity of MMP-2/9 (Hao et al., 2025)
	Glioblastoma	Nude mice	100 mg/kg, oral gavage	Significantly inhibit tumor growth; induce apoptosis of tumor cells	Activate the p53 pathway; induce G2/M phase arrest; inhibit VEGF expression (Jiang et al., 2021)
Inflammatory diseases	Collagen-induced arthritis	DBA/1J mice	40 mg/kg, oral gavage	Reduce joint swelling and inflammation; improve the pathological damage of joint tissues	Reduce the levels of pro-inflammatory factors in serum: TNF-α, IL-1β, IL-6; inhibit the activation of NF-κB pathway; suppress the expression of MMP-3 (Meng et al., 2021)
	Parkinson’s disease	MPTP-induced C57BL/6 mice	40 mg/kg, intraperitoneal injection	Improve motor dysfunction; protect dopaminergic neurons	Alleviating neuroinflammation: inhibiting microglial cell activation; Inhibiting oxidative stress: Increasing GSH, reducing MDA; Regulating the BDNF/TrkB signaling pathway (Xian et al., 2021)
Neurological disorders	Alzheimer’s disease	APP/PS1 transgenic mice	100 mg/kg, oral gavage	Improve cognitive function deficits; reduce Aβ plaque deposition	Inhibiting neuroinflammation: reducing the levels of TNF-α and IL-1β; regulating the expression of BACE1; activating the Nrf2 antioxidant pathway (Li et al., 2024a)

### 3.2 Combined drug use strategy

#### 3.2.1 Synergistic effect with chemotherapy drugs

As stated in Section 2.1.5, AE is a powerful chemical sensitizer, and its synergistic effects with standard chemotherapeutic drugs like cisplatin, doxorubicin, and paclitaxel are well-documented in the literature (Dai et al., 2025). This synergy is accomplished through various mechanisms: AE enhances the intracellular retention of chemotherapeutic agents by blocking efflux pumps like P-glycoprotein and reinstates the sensitivity of drug-resistant cancer cells to apoptosis through the modulation of p53 or Bcl-2 family proteins (Qu et al., 2025; Chen et al., 2023; He et al., 2023; Li X. et al., 2024). Simultaneously, the potent anti-inflammatory and antioxidant characteristics of AE can mitigate the severe side effects of chemotherapy, including organ damage from nephrotoxicity or cardiotoxicity, thus improving patients’ tolerance to treatment and potentially facilitating the implementation of more effective dosing regimens (He et al., 2023; Li X. et al., 2024).

#### 3.2.2 Combined application with immunotherapy

The integration of the immunomodulatory properties of AE with novel cancer immunotherapies constitutes a particularly promising domain. Immune checkpoint inhibitors, including antibodies that target PD-1 or PD-L1, have transformed cancer therapy (Yang et al., 2025); yet, their effectiveness is frequently restricted to patients with pre-existing T-cell infiltration within their tumors (Kim et al., 2025). The capacity of AE to alter the tumor microenvironment presents a compelling approach for transforming immunologically “cold” malignancies into “hot” cancers. By converting M2-type macrophages to the M1 phenotype, mitigating chronic inflammation, and potentially augmenting antigen presentation, AE might establish a more conducive tumor microenvironment for T-cell activation and invasion (Gao et al., 2025). This TME “reprogramming” is anticipated to synergize with ICIs, surmounting primary or acquired resistance and thereby broadening the patient pool that benefits from these groundbreaking medicines (Pich-Bavastro et al., 2023; Ye et al., 2024). This burgeoning field offers significant potential for future cancer therapy approaches.

### 3.3 Challenges in clinical translation

#### 3.3.1 Pharmacokinetics and bioavailability

The primary impediment obstructing the therapeutic advancement of AE is its inadequate “druglikeness,” particularly regarding its pharmacokinetic properties. A characteristic attribute of AE is its markedly low water solubility, which significantly limits the options for its formulations and its dissolving within the gastrointestinal tract (Ai et al., 2025; Xu et al., 2023). Following oral ingestion, it experiences significant first-pass metabolism in the liver and intestinal wall, primarily by glucuronidation and sulfation, and is swiftly digested (Ren et al., 2024; El-Say et al., 2023; Kumar et al., 2024). The synergistic impact of inadequate absorption and accelerated metabolism results in exceedingly low oral bioavailability, often ranging from 1% to 5%, complicating the attainment and maintenance of an effective plasma therapeutic dosage via standard oral administration (Xie et al., 2023; Sulistiawati et al., 2024).

AE shares many pharmacokinetic features with other plant-derived anthraquinones. Anthraquinones are absorbed mainly in the intestines, where free aglycones exhibit faster absorption than their glycosidic forms due to greater liposolubility; nonetheless, AE generally displays low oral bioavailability and considerable inter-individual variability (Wang et al., 2021). Anthraquinones frequently exhibit variable plasma concentration-time profiles characterized by several absorption peaks, owing to hepato-intestinal circulation, reabsorption, and dynamic interconversion among various anthraquinone molecules. Subsequent to absorption, they are extensively disseminated to tissues with abundant blood supply, such as blood, the gastrointestinal system, liver, lungs, kidneys, and adipose tissue. Significantly, AE can be transformed into other anthraquinones, including rhein and emodin, and may also originate from their metabolism, indicating that systemic exposure can be more accurately represented by its metabolites than by the parent substance. The primary excretion pathways for anthraquinones include renal, biliary, and fecal. The pharmacokinetic attributes-site-specific intestinal absorption, significant first-pass metabolism and interconversion, and swift excretion-elucidate the low or undetectable plasma levels of AE noted in clinical and preclinical studies, highlighting the necessity to assess both parent AE and its metabolites in evaluating efficacy and safety.

#### 3.3.2 Selectivity and off-target effects

The multi-target pharmacological properties of AE (Yan et al., 2023; Ouyang et al., 2024; Qu et al., 2025), specifically its capacity to engage with several targets, present a double-edged sword. This foundation for its success in complex, multifactorial disorders also raises questions regarding selectivity and potential off-target effects (Wang Z. et al., 2025; Ren et al., 2024). The absence of a singular, highly specific target presents obstacles for the design of pharmacodynamic biomarker studies and the prediction of its comprehensive biological effects (Ren et al., 2024; Dai et al., 2025). This “pan-targeting” may result in unforeseen side effects, particularly with prolonged use, complicating the safety profile necessary for clinical approval (He et al., 2023; Chen S. et al., 2025).

#### 3.3.3 Preclinical toxicity profile and limited clinical exposure

While typically regarded as safe at low dosages, particularly when ingested as a conventional herbal element, pure AE has demonstrated measurable hepatotoxicity and nephrotoxicity at relatively high experimental doses in certain investigations (Ren et al., 2024). The primary concerns are hepatotoxicity and nephrotoxicity, reported in animal models subjected to elevated doses (e.g., tens to hundreds of mg/kg/day of purified AE) or prolonged exposure (Li X. et al., 2024; Wang S. et al., 2025). The dose-dependent toxicity, coupled with the necessity of administering high dosages to offset its low bioavailability, results in a potentially small “therapeutic window”—the interval between the effective dose and the dangerous dose (Dai et al., 2025). Defining and sustaining drug exposure within this interval is a significant hurdle for its safe clinical application, necessitating meticulous dose exploration investigations and potentially therapeutic drug monitoring (Qu et al., 2025).

A systematic search of the Cochrane Library (search terms: “aloe-emodin”) did not identify any Cochrane systematic reviews or registered randomized trials focusing on aloe-emodin as a single pharmacological agent. Existing clinical data are therefore derived from small-scale trials and observational studies, many of which evaluate multi-component preparations containing AE rather than purified AE alone. These studies, including the pharmacokinetic investigation “Rhein and aloe-emodin kinetics from senna laxatives in man” (Krumbiegel and Schulz, 1993), which assessed clinically used doses of senna based laxatives in healthy volunteers, suggest potential utility in the context of laxative therapy but also highlight important limitations: aloe-emodin itself was not detectable in plasma at any sampling point, whereas its related anthraquinone rhein reached peak concentrations of approximately 150–160 ng/mL with biphasic maxima at 3–5 h and 10–11 h after dosing, likely reflecting absorption of free rhein and rhein released from prodrugs by colonic bacterial metabolism. These findings indicate that, under clinically used laxative regimens, systemic exposure to free AE appears to be minimal, and the observed effects are largely mediated by other anthraquinone derivatives or their metabolites. Overall, the clinical evidence remains preliminary and is limited by small sample sizes, heterogeneous study designs, and the difficulty of attributing efficacy and safety profiles specifically to aloe-emodin within multi-component herbal preparations.

## 4 Strategies for enhancing treatment outcomes

### 4.1 New drug delivery systems

Formulation science offers an effective arsenal to tackle the inadequate water solubility and decreased bioavailability of AE. Encapsulating AE within precise carrier systems enhances its stability, regulates its release profile, and enables targeted delivery to sick tissues (Xu et al., 2023; Yan et al., 2025). Nano-based techniques have emerged as the most promising strategy among these options. They employ liposomes to encapsulate AE within phospholipid vesicles, utilize polymer nanoparticles like PLGA for controlled and sustained release, and use self-assembled micelles as carriers (Song et al., 2024). These nano-platforms safeguard AE from early metabolic destruction, extend its *in vivo* circulation duration, and augment its accumulation at tumor locations via the “enhanced permeability and retention effect,” a passive targeting mechanism (Deshpande et al., 2023). Furthermore, the carriers’ surfaces can be altered with ligands for targeted delivery, so enhancing the drug’s efficacy at the site of action while minimizing systemic exposure (Bao et al., 2024; Khan et al., 2025). In addition to the nano-scale, other recognized formulation approaches, including solid dispersion technology that incorporates active ingredients in hydrophilic polymer matrices and the development of inclusion complexes with host molecules such as cyclodextrins, are extensively utilized (Xie et al., 2025). Both ways significantly enhance the dissolution rate and solubility of AE, offering more direct and beneficial strategies to augment its oral absorption and overall therapeutic effectiveness.

### 4.2 Chemical structure modification and prodrug design

As a complement to formulation strategies, medicinal chemistry offers a way to directly optimize the intrinsic properties of AE molecules themselves (Ai et al., 2025). Through rational chemical structure modification, the aim is to create new derivatives with enhanced drug-like properties (Hu et al., 2023; Ai et al., 2025). Such strategies include introducing hydrophilic groups to improve solubility, blocking key metabolic sites such as hydroxyl groups prone to glucuronidation to enhance metabolic stability, or fine-tuning the structure to increase binding affinity to specific targets (Guo et al., 2025). A particularly ingenious approach in this field is prodrug design, which temporarily masks the active AE molecule by attaching a chemical “precursor moiety”. This inactive prodrug form can be engineered to have superior properties, such as increased water solubility for intravenous injection or enhanced membrane permeability for improved oral absorption (Dai et al., 2025). Crucially, the precursor moiety is designed to be cleaved *in vivo*, ideally by enzymes overexpressed in the target tissue, thereby precisely releasing the active AE where needed. This dual-functional approach not only addresses pharmacokinetic challenges but also serves as an inherent targeting mechanism, significantly reducing systemic toxicity (Yan et al., 2025). Designing ester, phosphate ester, or glycosidic prodrugs of AE is currently being explored as promising avenues, with the potential to yield a new generation of AE-based therapeutic agents with clear and clinically translatable profiles.

## 5 Discussion

### 5.1 Future research directions

Notably, the effective concentrations and doses of AE required to modulate different pathways *in vitro* and *in vivo* are not directly comparable. In most cell-based studies, AE induces cell-cycle arrest and immunomodulatory/anti-inflammatory effects at approximately 5–20 μM^89^, whereas more stable inhibition of metastasis, angiogenesis and PI3K/Akt, MAPK or NF-κB signaling, as well as autophagy modulation, generally requires 10–40 μM. By contrast, in animal models, antitumor, anti-inflammatory or organ-protective effects are usually observed after administration of AE at roughly 10–100 mg/kg (Hu et al., 2021; Long et al., 2025), while pharmacokinetic data show that systemic plasma levels of the parent compound remain low and are often exceeded by those of its metabolites, reflecting poor oral bioavailability (Ai et al., 2025; Liu et al., 2025), extensive first-pass metabolism and interconversion within the anthraquinone family. This discrepancy suggests that some pathway-level effects seen only at high *in vitro* concentrations may not be fully achievable through direct systemic exposure *in vivo*; therefore, future studies should use *in vitro* concentrations closer to attainable *in vivo* exposure and define concentration-effect relationships for both AE and its major metabolites (Yao et al., 2024).

Notwithstanding considerable advancements in research, numerous fundamental scientific inquiries continue to be elucidated. At the fundamental mechanistic level, it is imperative to ascertain whether AE possesses one or more unidentified high-affinity core targets and whether its efficacy arises from the synergistic effects of multiple targets. This necessitates the utilization of advanced technologies, including chemical proteomics and single-cell multi-omics, to systematically delineate its molecular interaction network within specific pathological microenvironments and clarify the exact regulatory mechanisms governing its varied effects across different cell lineages. At the clinical translation level, advancements in these fundamental studies will establish a foundation for enhancing treatment tactics. The primary objective is to determine the ideal therapy window and recommended indications for AE, while identifying potential beneficiaries through dependable biomarkers. Furthermore, unique clinical studies utilizing biomarker stratification must be developed to rigorously assess their synergistic efficacy and safety as monotherapy or in combination, particularly with targeted and immunotherapies. At the pharmaceutical development stage, the primary requirement for achieving clinical value is to surmount critical technological obstacles. The primary challenge is to create unique drug delivery systems that exhibit high drug loading capacity, targeting specificity, biocompatibility, and scalability for production, consequently enhancing druggability and facilitating its advancement as a candidate drug.

### 5.2 Conclusion

Emodin derived from aloe is a naturally occurring active compound with significant potential for development. Its multifaceted pharmacological properties demonstrate significant potential in the treatment of important and complicated diseases, including cancers and inflammatory disorders. The transition from a natural substance to a therapeutic medicine encounters significant obstacles, including limited bioavailability and possible toxicity. However, these problems are not insuperable. The cross-integration of contemporary pharmaceutics, medicinal chemistry, and chemical biology has offered essential technical assistance in addressing these obstacles. By integrating and applying nanodelivery methods, prodrug design strategies, and precise structural alterations, we want to systematically transform this natural product into a new generation of precision therapeutic pharmaceuticals. Through ongoing interdisciplinary collaboration, this ancient phytochemical has the potential to be revitalized and offer novel answers to challenging clinical treatment issues.
